# Fermentation, fractionation and purification of streptokinase by chemical reduction method

**Published:** 2011-03

**Authors:** Z Karimi, M Babashamsi, E Asgarani, M Niakan, A Salimi

**Affiliations:** 1 Department of Biology, Faculty of science, Alzahra University, Tehran, Iran.; 2 Department of Immunochemistry, Monoclonal antibody Research Center, Avicenna Research Institute, ACECR, Tehran, Iran.; 3 Department of Medical microbiology, Shahed University, Tehran, Iran.

**Keywords:** Streptokinase, Fermentation, Purification, Chemical Reduction

## Abstract

**Background and Objectives:**

Streptokinase is used clinically as an intravenous thrombolytic agent for the treatment of acute myocardial infarction and is commonly prepared from cultures of *Streptococcus equisimilis* strain H46A. The objective of the present study was the production of streptokinase from strain H46A and purification by chemical reduction method.

**Materials and Methods:**

The rate of streptokinase production evaluated under the effect of changes on some fermentation factors. Moreover, due to the specific structure of streptokinase, a chemical reduction method employed for the purification of streptokinase from the fermentation broth. The H46A strain of group C streptococcus, was grown in a fermentor. The proper pH adjusted with NaOH under glucose feeding in an optimum temperature. The supernatant of the fermentation product was sterilized by filtration and concentrated by ultrafiltration. The pH of the concentrate was adjusted, cooled, and precipitated by methanol. Protein solution was reduced with dithiothreitol (DTT). Impurities settled down by aldrithiol-2 and the biological activity of supernatant containing streptokinase was determined.

**Results:**

In the fed –batch culture, the rate of streptokinase production increased over two times as compared with the batch culture and the impurities were effectively separated from streptokinase by reduction method.

**Conclusion:**

Improvements in SK production are due to a decrease in lag phase period and increase in the growth rate of logarithmic phase. The methods of purification often result in unacceptable losses of streptokinase, but the chemical reduction method give high yield of streptokinase and is easy to perform it.

## INTRODUCTION

Streptokinase (SK) is an extracellular protein produced by various strains of streptococci. Its activity was firstly reported by Tillet and Garner, 1933 (1) who discovered the haemolytic activity of this protein. It is now well established that the fibrinolytic activity of streptokinase originates in its ability to activate plasma plasminogen (2, 3). Streptokinase is one of the two protein components of the thrombolytic agent known as APSAC (anisoylated plasminogen streptokinase activator complex) described in EP-A-0028489.

Streptokinase is produced by certain Streptococci and certain bacteria which contain appropriate genetic material derived from Streptococci of Lancefield groups A, C or G. Streptokinase which is to be used for clinical purposes is commonly prepared from cultures of *Streptococcus equisimilis* strain H46A, from which the secretion of streptokinase into the external medium is directed by a 26 amino acid signal peptide which is cleaved during the secretion process. The mature protein has a molecular weight of about 47 kilo Dalton (kD) and was found to be composed of 415 amino acid residues (4, 5). The growth of a β-hemolytic streptococcus was studied in continuous culture with pH as a limiting factor (6, 7). In these experiments, pH was controlled only by addition of buffer to the medium. The yield of cells and of some extra cellular antigens was investigated. Rosenberger and Elsden (8) studied the effect of both glucose and tryptophan limitation on growth in continuous cultures of a *Streptococcus faecalis* strain.

Numerous methods of purifying streptokinase have been described which are based on quantitative differences in solubility, electrical charge, molecular size and shape or non specific physical interactions with surfaces (9, 10). Recently we have produced a fusion recombinant streptokinase and purified it in a single step affinity chromatography using glutathione as the ligand (11) and by affinity chromatographatography on acylated plasminogen with ?-nitro phenyl guanidinobenzoate (NPGB) (12). Unlike the contaminating proteins which make up the impurities, such as streptolysin or streptodornase in a culture product of H46A, streptokinase is a single chain protein that does not contain the amino acids cysteine or cystine (13, 14). This structural difference was employed to provide a method for the purification of streptokinase from the fermentation broth.

## MATERIALS AND METHODS

The bacterial strain and materials used in the present study includes; *S. equisimilis* group C, strain H46A (ATCC 12449, USA), Todd Hewitt Broth (THB, HiMEDIA Laboratories), Brain Heart Infusion (BHI, USA), Trypticase Soy Agar (TSA, BBL, USA) Lysine monohydrochloride (Sigma Chemical, USA), Hexyl resorcinol (Merck, Germany), 3-amino-n-caproic acid (EACA, Sigma Chemical, USA), Cyanogen bromide-activated Sepharose 4B (Sigma Chemical, USA), Chromogenic substrate S-2251 (Chromogenix laboratories, Italy), Dithiothreitol (Sigma Chemical, USA), Aldrithiol-2 (Sigma Chemical, USA), Salts, acids and bases for buffers (Merck, Germany).

**Fermentation and fractionation**. The strain H46A was cultured in TSA at 37°C. In two separate flasks, one of the colonies was grown in 10 and 100 ml of Brain Heart Infusion (BHI) at 37°C. By increasing the turbidity to the level of OD=0.6 at 600 nm, it was transferred separately to 1 liter vessel of a BioFlo 110 fermentor. The turbidity and SK activity measured by 2 hours intervals for both cultures. As a result of improved SK activity for 10% inoculation at 6 hours, the culture repeated for 10% inoculation in five separate batch cultures and pH maintained from 5-9 by acetic acid and NaOH. A higher SK activity observed at condition of neutral pH. To improve the growth condition, the pH of a fed batch culture was maintained at 7 during incubation at 37°C for 10 hours by addition of sterile 4% (w/v) glucose and 5.0 N NaOH. The activity of secreted streptokinase was determined by solid and liquid colorimetric methods. The fermentation was stopped by rapid cooling to 4°C and by the addition of hexyl resorcinol. The culture was centrifuged for 25 minutes at 10,000 g and the supernatant was filtered through a 0.2 µm cellulose acetate filter. The filtrate was concentrated by ultrafiltration. The pH of the concentrate was adjusted, cooled, and the fraction precipitating by 30% cold methanol was harvested. This was dissolved in water at neutrality.

**Fig. 1:**
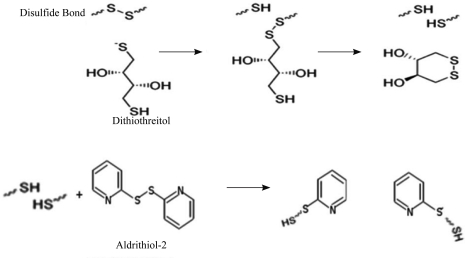
Mechanism of protein reduction and separation.

**pretreatment of streptokinase-containing protein solution**. The mechanism of protein reduction and separation is chemically explained in (Fig. 1). Protein solution was reduced with 100 mM DTT and incubat- ed at 30°C for 30 minutes. Aldrithiol-2 to a final concentration of 150 mM was then added and the solu-tion (at pH 7.5)incubated with agitation for 20 minutesat 30°C followed by 15 minutes at 35°C. The solution was then cooled to 5°C and held for 20 minutes

**Removel of impurity and SDS-PAGE analysis**. Residual Aldrithiol-2 and precipitated protein contaminants were removed by centrifugation at 6000g for 20 minutes at 5°C. The supernatant was then decanted from the precipitate. The purity of the streptokinase was evaluated by mixing 20 µl of the supernatant in 50 µl of 1x SDS sample buffer containing (Tris base 7.5%, 2 ml SDS 10%, 1 ml Glycerol, 2 mg Bromophenol blue, 25 µl 2 ME), heated at 100°C for 3 minutes, spinned down and the supernatants were applied to a 12% SDS- Poly Acrylamide Gel Electrophoresis. The gels were stained with Coomassie blue, and the protein bands were visualized by destaining with methanol-acetic acid solution.

**Determination of streptokinase activity**. The activity can be determined by solid or liquid plasmin hydrolysis of chromogenic peptidyl anilide substrate (S-2251) methods (15, 16). In a total volume of 131.5 µl containing 1.5 µl of 50 mM Tris-HCl (pH 7.4) and 30 µl of plasminogen (0.2 mg ml^-1^) the reaction was initiated by adding of the 15µl sample of extracted streptokinase, incubated for 15 minutes at 37°C and added 75 µl of S-2251 substrate (5 mg ml-1), again incubated for further10 min at 37°C.

The reaction was stopped by adding 10 µl of acetic acid (0.4 N) and monitored at 405 nm.

## RESULTS

Bacterial cells proliferated logarithmically in the first four hours and after that, the rate of proliferation decreased. It means bacterial cells are being entered to the stationary phase, this condition extended to the 8th hour. Streptokinase production increases near-by three fold with use of 10% inoculum, adding glucose and adjusting pH simultaneously (Table 1).

**Table 1:** Bacterial growth at 600 nm and SK activity (U/L) at different inoculations, pH adjustment and glucoseaddition.

Time/hours	t0	t2	t4	t6	t8	t10
Turbidity (1% inoculum)	0.03	0.1	0.2	0.4	0.6	0.67
Turbidity (10% inoculum)	0.1	0.3	0.6	0.65	0.68	0.7
Turbidity (10% inoculum & pH regulation)	0.1	0.1	0.4	0.64	0.56	0.54
Turbidity (10% inoculum, pH regulation & glucose addition)	0.1	0.56	0.89	0.925	0.925	0.925
SK/U (1% inoculum)	874	300000	370000	1100000	2250000	1900000
SK/U (10% inoculum)	300000	750000	2250000	2400000	2100000	1900000
SK/U (10% inoculum & pH regulation)	300000	1050000	2250000	2650000	2650000	2650000
SK/U (1% inoculum, pH regulation & glucose addition)	300000	2250000	5600000	6000000	5600000	4500000

**Fig. 2:**
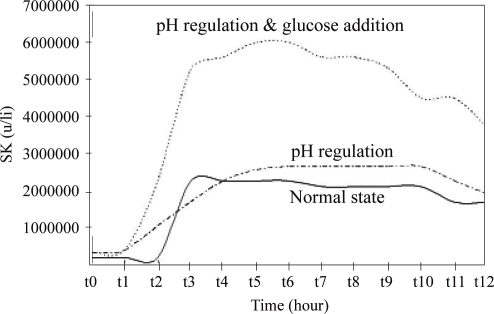
Streptokinase change in culture by pH regulation and glucose addition.

The bacterial strain H46A produced relatively high yields of streptokinase by 10% inoculation, pH adjustment and glucose feeding. The rate of streptokinase secretion increased significantly in condition of excess glucose addition (40 grams/L) to culture media. As a result of glucose metabolism, acid was produced. So, the acidity neutralized by NaOH, when the pH in the culture was controlled within the range of 7.0 to 7.1.the activity of streptokinase increased (Table 2 & Fig. 2).

**Table:** SK activity in a 6 hours culture by pH regulation.

pH	5	6	7	8	9
**SK/U (10%inoculum)**	2130000	2225000	2650000	2200000	1700000

**Fig. 3:**
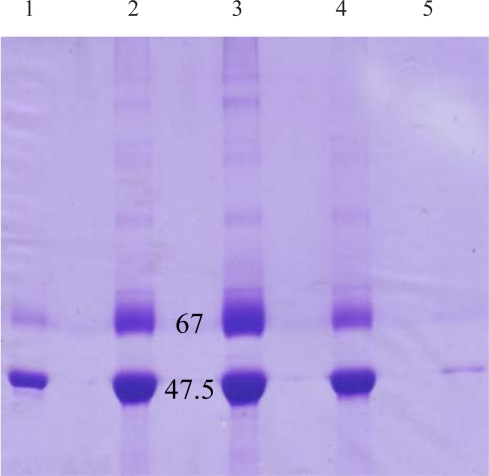
SDS-PAGE: Purification of crude SK (different concentrations) with 100 mM DTT.(lane 1); 2 mg crude SK. (lane 2); 5 mg crudeSK. (lane 3); 8 mg crude SK. (lane 4); 6 mg crude SK. (lane 5); Standard SK.

**Fig. 4:**
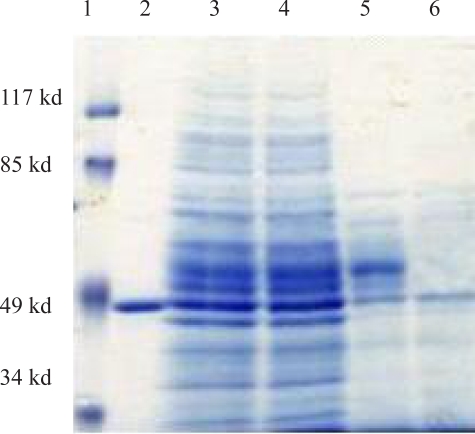
SDS-PAGE: Purification of SK from H46a culture by reducing agent(lane 1); Protein MW Marker. (lane 2); Standard SK. (lane 3& 4); H46a culture. (lane 5); 2 mg methanol extract treated with 200 mM DTT & Aldrithiol. (lane 6); 2 mg methanol extract treated with 300 mM DTT & Aldrithiol

The purification steps of commercial partial purified streptokinase ( Heberkinasa) and methanol extract of H46a are shown in (Figs. 3 & 4).

## DISCUSSION

The findings of Rosenberger and Elsden (8) indicate that, to obtain maximal cell yield per unit energy source, the energy source should be the limiting factor. Studies on the growth of a β-hemolytic streptococcus by Carush et al, (6) revealed that pH is a limiting factor. This study supports the findings of Dubey et al, (17) regarding to pH sensitivity of streptokinase. The comparison of this study with other studies shows that, simultaneous adjustment of pH, inoculation rate and proper glucose feeding could produce relatively high yields of streptokinase. Because of high sensitivity of streptokinase to pH (The optimum pH for cell growth and streptokinase production is 7) and temperature change, some part of this product is degraded at the same time. Therefore, conditions that decrease lag phase period and increase the growth rate of logarithmic phase, results in higher SK yield. By adjustment of culture period, glucose feeding and pH maintenance with concentrated NaOH the rate of product increased up to three times. Moreover, by using suitable amount of hexyl resorcinol, the probable infection with the pathogenic streptococcus during the process was prevented.

Numerous methods have been reported for the purification of streptokinase obtained form the culture media of various strains of streptococci. (18–20, 13). In some cases DEAE-cellulose has been used in combination with other purification procedures and a highly purified product has been obtained. Other chromatographic procedures have also been used for the purification of streptokinase by combining more than one purification step are often result in unacceptable losses of streptokinase or inadequate removal of impurities and employ expensive harsh or flammable reagents. Several affinity chromatography methods utilizing plasmin or plasminogen as ligand, have been discussed for purifying streptokinase (21–25). Purification through the affinity column caused a 30% decrease in the streptokinase activity.

Streptokinase, unlike the contaminating proteins which make up the impurities, does not contain the amino acids cysteine or cystine.

In conclusion this structural difference employ- ed to provide a more effective method (Chemical Reduction Method) for the purification of streptokina- se from the fermentation broth. The mentioned method dose not require the expencive affinity columns or multi steps purification facilities. Moreover, this method gives high yield of streptokinase and is easy to perform it.
